# Practical Remarks

**Published:** 1885-02

**Authors:** C. Hering


### PRACTICAL REMARKS.

Cases spoiled by the use of Aconite may often be retrieved by use of Sulphur.

Arnica is more apt to spoil a case than Aconite. Arnica makes a much more profound impression upon the system than Aconite. Its real culminating action is similar to typhus fever. Brilliant results have frequently been obtained from it in the worst forms of typhus.

Physicians who wear spectacles, and who have to ride long distances in very cold weather, will find protection from freezing of the parts coming in contact with the metal by bathing the skin with Camphor.

Ranunculus bulb. is one of our most effective agents for the removal of bad effects of the abuse of intoxicating drinks.

"At least one-half of the chronic diseases of women and children are developed by using too much sugar."

Aconite is one of the most frequently indicated remedies, when the development of the organic disease of the heart manifests itself by tingling in fingers, numbness and lameness of left arm.—*C. Hering.*

